# Peutz-Jeghers Syndrome: A Very Rare Cause of Iron Deficiency Anemia

**DOI:** 10.4274/tjh.galenos.2018.2018.0164

**Published:** 2019-02-07

**Authors:** Fatma Demir Yenigürbüz, Ugur Deveci, Ebru Tuncez

**Affiliations:** 1 Şanlıurfa Training and Research Hospital, Clinic of Pediatric Hematology, Şanlıurfa, Turkey; 2 Şanlıurfa Training and Research Hospital, Clinic of Pediatric Gastroenterology, Şanlıurfa, Turkey; 3 Şanlıurfa Training and Research Hospital, Clinic of Medical Genetics, Şanlıurfa, Turkey

**Keywords:** Peutz-Jeghers syndrome, Iron deficiency anemia, Bleeding

An 11-year-old boy was admitted to the clinic with a 1-year history of fatigue and abdominal pain. In physical examination, there were multiple small, flat brown-violet pigmentations on his buccal mucosa and lips and brown spotty pigmentations on the lip mucosa, present since his birth (Figure 1). His mother also had similar mucocutaneous pigmentation and was operated on for intestinal polyps (Figure 2).

Laboratory findings were consistent with severe iron deficiency anemia and the fecal occult blood test was positive. Gastrointestinal endoscopic examination revealed two polyps of the stomach and three polyps of the jejunum that caused bleeding were removed with forceps (Figure 3). 

Histopathologic examination revealed hamartomatous polyps. The presence of brown pigmentations and multiple gastrointestinal polyps alerted us to a possible diagnosis of Peutz-Jeghers syndrome and serine/threonine kinase 11 (STK11, also called LKB1) mutation was found positive in both the patient and his mother. 

It is very important to conduct a thorough physical examination and to probe the family history in cases of iron deficiency anemia that is frequently encountered in children, especially in the presence of other complaints such as abdominal pain [1,2]. This allows early diagnosis of rare diseases such as Peutz-Jeghers syndrome, which leads to a high risk of developing cancer, and examination of family members for the associated complications by using advanced diagnostic tools [3,4,5].

## Figures and Tables

**Figure 1:**
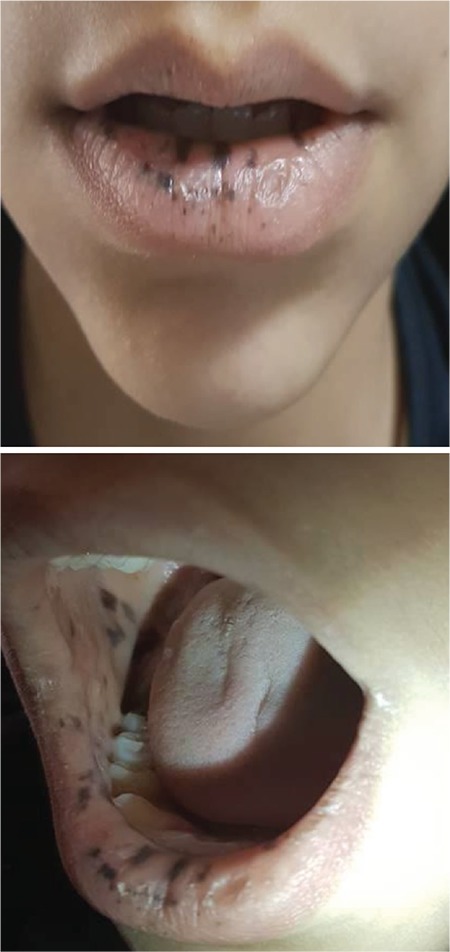
Multiple small, flat brown-violet pigmentations on the patient’s buccal mucosa and lips and brown spotty pigmentations on the lip mucosa.

**Figure 2:**
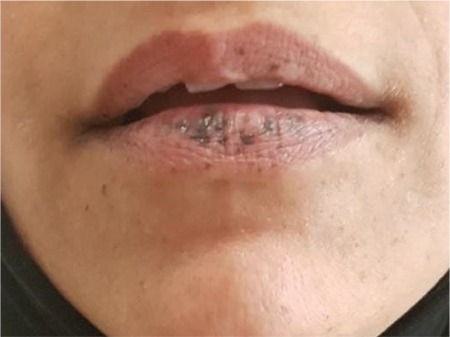
Similar mucocutaneous pigmentation of the patient’s mother.

**Figure 3:**
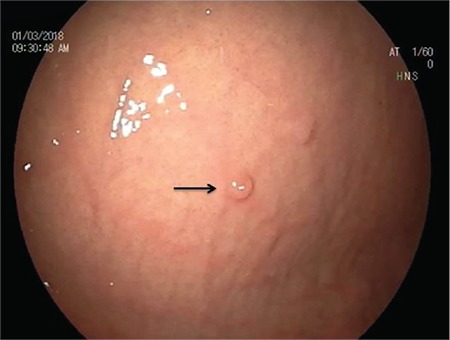
Polyps were revealed in the patient’s gastrointestinal endoscopic examination.
